# Computational mechanisms of belief updating in relation to psychotic-like experiences

**DOI:** 10.3389/fpsyt.2023.1170168

**Published:** 2023-05-05

**Authors:** Sophie Pauline Fromm, Lara Wieland, Arne Klettke, Matthew R. Nassar, Teresa Katthagen, Sebastian Markett, Andreas Heinz, Florian Schlagenhauf

**Affiliations:** ^1^Department of Psychiatry and Neuroscience | CCM, NeuroCure Clinical Research Center, Berlin Institute of Health CCM, Charité-Universitätsmedizin Berlin, Freie Universität Berlin, Humboldt-Universität zu Berlin, Berlin, Germany; ^2^Charité – Universitätsmedizin Berlin, Einstein Center for Neurosciences Berlin, Berlin, Germany; ^3^Bernstein Center for Computational Neuroscience, Berlin, Germany; ^4^Department of Psychology, Humboldt-Universität zu Berlin, Berlin, Germany; ^5^Carney Institute for Brain Science, Brown University, Providence, RI, United States; ^6^Department of Neuroscience, Brown University, Providence, RI, United States

**Keywords:** reward learning, belief updating, uncertainty, psychotic-like experience (PLE), online study, precision

## Abstract

**Introduction:**

Psychotic-like experiences (PLEs) may occur due to changes in weighting prior beliefs and new evidence in the belief updating process. It is still unclear whether the acquisition or integration of stable beliefs is altered, and whether such alteration depends on the level of environmental and belief precision, which reflects the associated uncertainty. This motivated us to investigate uncertainty-related dynamics of belief updating in relation to PLEs using an online study design.

**Methods:**

We selected a sample (*n* = 300) of participants who performed a belief updating task with sudden change points and provided self-report questionnaires for PLEs. The task required participants to observe bags dropping from a hidden helicopter, infer its position, and dynamically update their belief about the helicopter's position. Participants could optimize performance by adjusting learning rates according to inferred belief uncertainty (inverse prior precision) and the probability of environmental change points. We used a normative learning model to examine the relationship between adherence to specific model parameters and PLEs.

**Results:**

PLEs were linked to lower accuracy in tracking the outcome (helicopter location) (β = 0.26 ± 0.11, *p* = 0.018) and to a smaller increase of belief precision across observations after a change point (β = −0.003 ± 0.0007, *p* < 0.001). PLEs were related to slower belief updating when participants encountered large prediction errors (β = −0.03 ± 0.009, *p* = 0.001). Computational modeling suggested that PLEs were associated with reduced overall belief updating in response to prediction errors (β_*PE*_ = −1.00 ± 0.45, *p* = 0.028) and reduced modulation of updating at inferred environmental change points (β_*CPP*_ = −0.84 ± 0.38, *p* = 0.023).

**Discussion:**

We conclude that PLEs are associated with altered dynamics of belief updating. These findings support the idea that the process of balancing prior belief and new evidence, as a function of environmental uncertainty, is altered in PLEs, which may contribute to the development of delusions. Specifically, slower learning after large prediction errors in people with high PLEs may result in rigid beliefs. Disregarding environmental change points may limit the flexibility to establish new beliefs in the face of contradictory evidence. The present study fosters a deeper understanding of inferential belief updating mechanisms underlying PLEs.

## Introduction

Conceptualized as the “extended psychosis phenotype,” psychotic phenomena presumably exist on a dimension, ranging from manifest psychosis to mild subclinical psychotic-like experiences (PLEs) (1). PLEs involve unusual subjective experiences that resemble subtle psychotic symptoms but do not necessarily cause distress (2). PLEs are not uncommon among the general population (3, 4) and may be grounded in similar underlying cognitive and neurobiological mechanisms as manifest psychotic symptoms (5). Theoretical accounts of psychotic experiences suggest alterations in belief updating processes as underlying mechanisms, which can be described in a Bayesian framework of belief updating (6–10). In this framework, new evidence is constantly integrated into prior beliefs to minimize prediction errors (PEs) and to optimally predict future states (6, 7). Crucially, the degree to which some new sensory evidence updates prior beliefs to posterior beliefs depends upon the precision afforded by the sensory evidence and prior beliefs. Precision can be regarded as a measure of reliability or certainty about prior beliefs or sensory evidence. New observations will more influentially update the prior belief if they are considered reliable, meaning that the observer is very certain about this new piece of evidence. On the other hand, new evidence will be less influential if the observer is very certain about the prior belief. Consequently, the observer must accumulate sufficient new evidence (e.g., sustained and large prediction errors) to revise the prior belief. While the former strategy may lead to belief instability, the latter may cause belief rigidity. Thus, in the setting of evidence accumulation and belief updating, the precision ratio (inverse belief uncertainty) determines the learning rate. Our analysis below leverages this intimate relationship between learning rates and prior precision. Recent studies report altered integration of prior beliefs and new evidence in people on the subclinical and clinical psychosis spectrum (10, 11). However, there are mixed results regarding the directionality, with some studies suggesting over-updating of beliefs in patients with schizophrenia (12, 13) and others in belief perseverance (14, 15). Novel change point detection paradigms highlighted that patients with schizophrenia show a mixture of both over-updating and perseveration of beliefs (16). Patients with schizophrenia seemed to update beliefs in an “all or nothing” manner. When belief uncertainty was high, they updated their beliefs completely (“all”), instead of moderately integrating new evidence into the prior (16). During other times, they perseverated on the prior belief (“nothing”). This strategy hampers the balanced integration of new and old information, thereby limiting belief-flexibility and -precision. Research on the *jumping-to-conclusion* bias also suggests that patients with schizophrenia rely more heavily on initial observations, quickly manifest beliefs, and fail to keep integrating later information (15, 17). These findings have in common that the weighting of evidence and prior differed between control participants and patients as a function of belief uncertainty. As an example, in clinical delusions, this could manifest as a person quickly adapting, e.g., a paranoid belief during an initial state of belief uncertainty and consequently sticking to this belief with high certainty despite conflicting evidence. Multiple accounts suggested that not only updating toward the precision of the outcome belief plays a role in psychosis but also the adaptation toward the precision of the environment. As such, previous studies showed that patients with schizophrenia (18) or a first episode of psychosis (19, 20) and healthy people with high schizotypy (20) adapt learning signals less toward the precision of the environment. Similar alterations pertaining to belief updating behavior have been reported in other psychiatric disorders (21). This includes obsessive–compulsive disorder (22, 23) or anxiety and depression (24, 25) and opens up the debate as to whether these alterations can be considered as a mechanism that is specific to psychotic experiences.

The present study aimed to examine belief updating in the general population and explore possible associations with PLEs. We administered a previously established feedback-driven change point detection task (16, 26–29). Although variants of these tasks have been used successfully as online versions (30), helicopter gamification for the first time is presented here as an online study. In this paradigm, optimal learning requires updating the estimate (belief) of a hidden helicopter's location. The helicopter usually stays in one place and drops bags whose exact location noisily oscillates around the helicopter (uncertainty). The helicopter sometimes changes location completely (environmental change point), which introduces unexpected uncertainty in the paradigm. Thus, the task requires participants to integrate the uncertainty about the helicopter location relative to the noise in the outcome process and the unexpected uncertainty due to sudden change points. Upon these change points, participants have to disregard their prior belief of the helicopter location and quickly learn the new position. The learning rate should increase (1) when an environmental change point occurred and (2) when the observer is uncertain about their current belief. Belief uncertainty about the next bag drop depends on the contextual noise, i.e., how much the bag locations vary around the helicopter's position, and on the relative uncertainty that decreases with the number of observations since the last change point. We investigated how PLEs relate to feedback-driven learning in different noise contexts and examined how participants integrate change point probability and uncertainty within each context. To the best of our knowledge, there is no gold standard to assess PLEs comprehensively in the general population. Therefore, we used three different questionnaires (31–33) to assess PLEs and calculated a composite score to combine these measures. Finally, we probed the relationship of model parameters of change point probability and uncertainty with self-reported obsessive–compulsiveness, anxiety, apathy, and alcohol use to test for the specificity of our results with PLEs.

## Methods

The present project and analysis strategy were preregistered in the Open Science Framework (DOI 10.17605/OSF.IO/E8UXC).

### Participants

The sample size was oriented toward a prior online study with similar tasks and hypotheses (30). We adopted a linear multiple regression approach, where a sample size of n = 300 allowed us to detect small effect sizes of *f*^2^ = 0.058 (34) with a power of 95% at a confidence level of 95%. Sampling was terminated as soon as *n* = 300 datasets that fulfilled inclusion criteria were collected. Exclusion criteria involve incompleteness of questionnaire data, failure of more than three attention checks, and missing task data that exceeds 25% of the trials per run. Single trials in which participants were not attentive, indexed by reaction time larger than 3 s after the target were excluded as well. Recruitment took place *via* the online platform Prolific (www.prolific.co [accessed 11–20 November 2021]), which provides a means to recruit people who are interested in participating in online studies. Participants were prescreened for age (between 18 and 60 years) and fluency in English. Participants were reimbursed financially (£7,50/hr) *via* Prolific after study completion.

### Procedure

First, all participants were presented with the study information and the consent forms. Upon completion, they were asked to provide non-identifying demographic data, followed by the belief updating task hosted in JATOS (35). Next, they were asked to complete self-reports hosted in the clinical data management software RedCap at Charité (36) and finally, they were allowed to report problems during the study. The study procedures received approval from the ethics committee at Charité Berlin, and all online procedures were conducted in compliance with the guidance of the data protection committee *(*EA2/156/18*)*.

### Measures

#### Psychiatric questionnaires

Measures of PLEs included Peters Delusion Inventory (PDI) (25), Cardiff Anomalous Perceptions Scale (CAPS) (26), and the Aberrant Salience Inventory (ASI) (27). We used these complementary measures to derive a comprehensive assessment of PLEs for the general population. Scores of all PLE measures were z-scored and summed up to compute individual PLE sum scores. Since the PLE score comprises overlapping constructs covered by the three questionnaires (PDI, ASI, and CAPS), we conducted a factor analysis to distinguish and cluster the items of all questionnaires into coherent scales, allowing us to examine the subcomponents of PLEs and their relationship with belief updating more closely (details in Supplementary material). To test for the specificity of our results, we collected self-reports of the Obsessive-Compulsive Inventory-Revised (OCI-R) (37), State-Trait-Anxiety-Inventory (trait scale) (38), Apathy Evaluation Scale (AES) (39), and the Alcohol Use Disorder test (AUDIT) (40). Data quality was ensured by the implementation of attendance checks in multiple questionnaires.

#### Belief updating task

**Figure 1:**
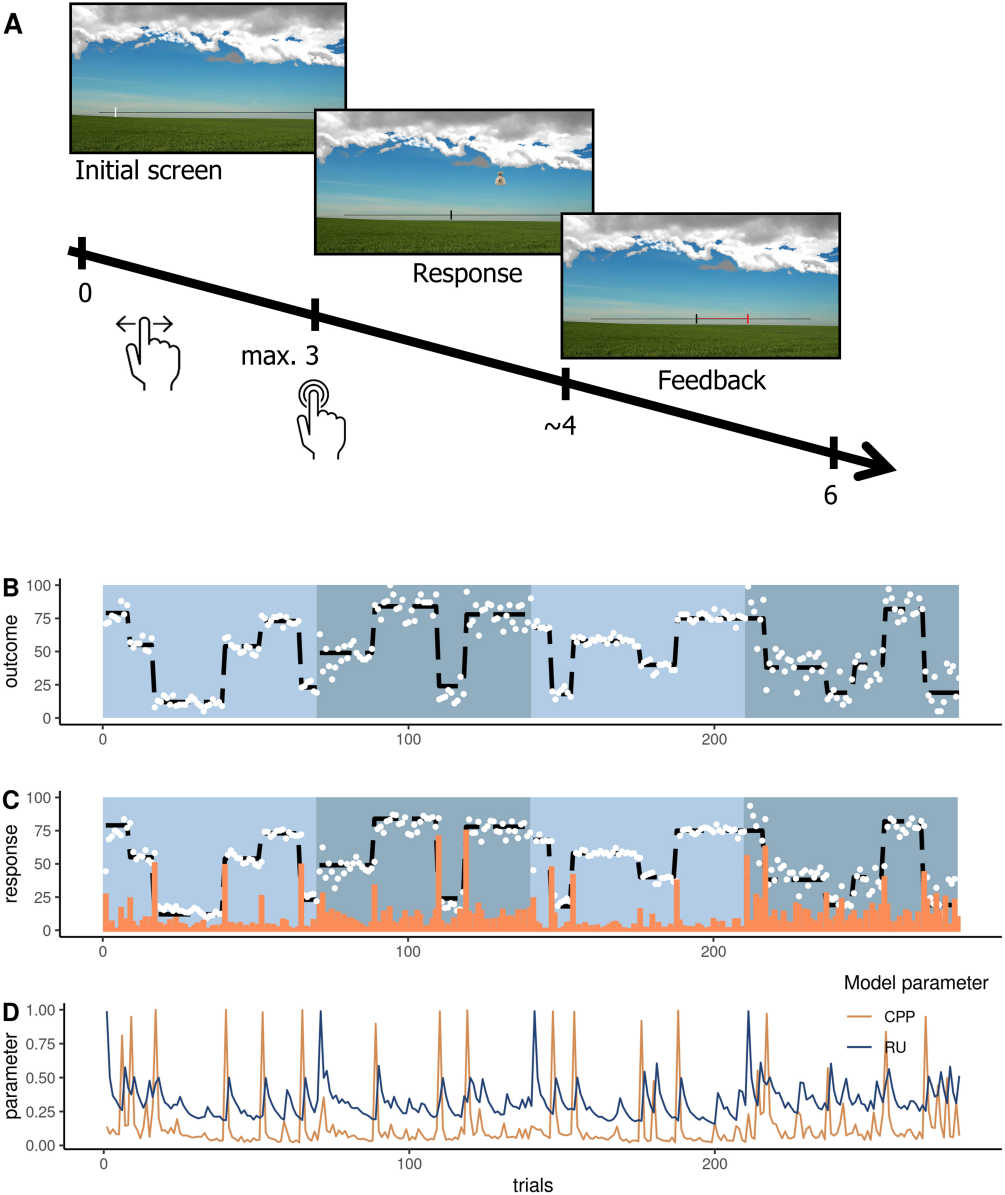
Belief updating task where participants place a bucket (response) to catch dropping bags and receive feedback about the deviation between their belief and the actual outcome (bag drop location). **(A)** Depiction of a trial with a timeline in seconds from the trial onset. **(B)** The trajectory of the helicopter location with the outcomes (white dots) and underlying mean (black dashed line) for the four runs. Blue shaded blocks represent the four runs. **(C)** Responses were averaged across subjects (white dots), the underlying mean of the outcome (black dashed line), and average absolute prediction errors (orange bars). **(D)** The trajectory of change point probability (CPP in orange) and relative uncertainty (RU in blue) was derived from the computational model approximating optimal Bayesian learning and averaged across subjects (16).

We administered an established feedback-driven belief updating task (16, 27, 28, 41) for the first time presented as an online study. In this paradigm (Figure 1), participants are instructed to place buckets on a scale via a button press to catch bags falling from a hidden and moving helicopter. In order to perform accurately, they are thus required to track the location of the helicopter (belief), which noisily oscillates around a certain spot (uncertainty) and sometimes changes location completely (change point). To indicate their current belief about the helicopter's position, participants were instructed to click on a scale, which initiated the bag drop (Figure 1A). The bags that dropped from the helicopter were either blank or marked with a € sign. Blank bags were introduced as containing no monetary reward for the participant, whereas € bag would result in a monetary reward after the game to incentivize accuracy. Which bag the helicopter dropped was unknown to the participant and was only revealed after participants indicated their belief of the helicopter location. The bag drop was followed by feedback on how far the prediction deviated from the actual helicopter location (PE). Then, the feedback vanished and a new trial started with the mouse cursor initialized at the location of choice of the previous trial. All participants observed the same outcomes (location of the bag drops) (Figure 1B). Outcomes were sampled from a normal distribution with a mean ranging from 0 to 100 and fixed variance. The mean was randomly reset with a chance level of .125 and represents the probability of change points (often termed as hazard rate). We manipulated environmental precision by altering the level of “noise” in the variation of the helicopter around the underlying mean. Therefore, we altered the variance of the generative distribution from which we sampled the helicopter's location. This variance was either low (SD = 3.33 units) or high (SD = 8.33 units). Participants completed two high-noise and two low-noise runs, each comprising 70 trials (a total of 280 trials). The order of runs was randomized across participants. Each run consisted of 4–6 stable phases that were segregated by change points. Our main outcome variable was participant's bucket placements that they logged in on the horizontal scale of the screen.

### Analysis

The present study comprises three main analyses: (1) Raw data analysis of observed behavior that is directly calculated from the participant responses. (2) Computational modeling of Bayesian learning, where we ran a normative model on the observed PEs of each participant to infer individual trial-wise estimates of change point probability (CPP) and relative uncertainty (RU). We then predicted individual trial-wise belief updates by these Bayesian model trajectories (CPP and RU) with linear regression to examine to what extent belief updates are informed by CPP and RU. (3) Associations between belief updating and PLEs, where we used indices of belief updating calculated from (1) and (2) and probed associations with PLEs.

#### Raw data analyses of observed behavior

We computed trial-wise PEs (δ_t_) by subtracting the true position where the bag dropped (X_t_) from the participant's prediction (B_t_) (formula 1) and performance error by subtracting the helicopter location (underlying mean of the sampling distribution) from the participant prediction. We computed learning rates by dividing the belief update of the current trial by the PE of the previous trial (formula 2). These quantify learning speed and describe how much participants update their beliefs relative to the prediction error. Learning rates larger than 1 were rounded to 1 and learning rates smaller than 0 were rounded to 0. Learning rates scale the PE to determine how the evidence is weighted against the prior belief to form the posterior belief (B_t+1_) (formula 3).


(1) $$\begin{array}{l} \delta_{t}=X_{t}-B_{t}\; \end{array}$$

(2) $$\begin{array}{l} \alpha_{t}=\frac{-\left(B_{t+1}-B_{t}\right)}{\delta_{t-1}}\; \end{array}$$

(3) $$\begin{array}{l} B_{t+1}=B_{t}+\alpha_{t}^{*}\delta_{t}\; \end{array}$$

In order to check whether participants understood and performed the task as intended, we investigated how performance errors evolved across trials after change points (TAC) in the low and high noise conditions using a linear mixed regression model (details in Supplementary material). Crucially, we added PLE to this model to investigate the relationship with performance. All terms (TAC, noise, and PLE) were allowed to interact with each other. Additionally, we specified a random intercept for *subjects* and random slopes for TAC, noise, and the interaction of both terms. We then compared the full model to all reduced model versions and evaluated the best-fitting model using the buildmer function from the R-toolbox. In the results section, we report the best converging model. The best model was determined according to a likelihood-ratio test (LRT) based on chi-square mixtures to test for the contribution of terms to model fit, which is the default in the “buildmer” toolbox. Additionally, we report model selection based on the Akaike Information Criterion (AIC) in Supplementary material.

In an exploratory analysis, we probed the association between PLEs and learning rates in trials with high and low absolute PE magnitudes. Thereby, we tried to delineate whether learning speed is differentially related to PLE for small and large PEs. To this end, we aggregated learning rates per subject for trials when participants observed large absolute PEs (PE > 50) or small absolute PEs (PE < 5). We then set up a regression model for each category to predict average learning rates per participant by PLEs.

Next, we computed a raw data measure of belief precision using a previously outlined method (16) to describe how participants integrate observations across time. In theory, the precision of beliefs increases as participants integrate new and old information by using moderate or small learning rates, remains the same if participants stick to their previous belief, and resets to 1 if participants completely update beliefs to reflect the most recent outcome. For each trial, precision is computed as a ratio of the influence of the most recent observation on the present belief, as compared to the previous observations. After a change point, precision should first drop since only the most recent observation can accurately inform the belief. Then it should increase across subsequent observations as they are integrated into the belief. As soon as a learning rate of 1 occurs, the previous samples do not play into the current belief and precision goes back to 0. The number of previous observations in combination with the variance of the weighted sum constitutes a measure of precision (for the formula, see Supplementary material). To analyze how precision evolves across TAC, noise conditions, and whether this relates to PLE, we used the same linear mixed regression model for performance errors described above and in Supplementary material.

#### Computational modeling of optimal Bayesian learning

To formalize optimal belief updating in our paradigm, we applied the normative model that approximates optimal Bayesian learning as implemented (16) and described in previous work (26, 42). This model updates beliefs via PEs that are weighted by a learning rate (formulas 1–3). Unlike in the raw data analyses, the learning rate of the computational model α_t_ is adjusted according to trial-wise estimates of relative belief uncertainty (RU) and change point probability (CPP). If a large PE occurs, CPP approaches 1 indexing that the most recent observation is likely generated after a meaningful environmental change. RU is also updated on every trial and evolves as an estimate of the number of trials since the last change point—it decreases as more observations are made. Together these factors determine the learning rate α_t_ in the normative model (formula 4).


(4) $$\begin{array}{l} \alpha =CPP_{t}+RU_{t}+CPP_{t}\;*\;RU_{t} \end{array}$$

The optimal learner should scale the influence of a PE according to CPP and RU to optimally update their expectation and eventually maximize rewards. An example trajectory of these parameters is shown in Figure 1D. Intuitively, as long as observations are sampled in a stable environment, CPP is low, uncertainty slowly decreases, and beliefs become more precise. As soon as a large PE occurs, CPP peaks and RU increases subsequently. This scales up the learning rate so that rapid adaptations in belief updating as a response to a change point are possible. For each participant, we applied this model to the individual observed behavior and feedback (PEs and belief updates) to compute individual estimates of CPP and RU. To investigate how participants integrate PEs in the formation of new beliefs, we set up individual linear regression models (all regressors mean-centered) to predict each participant's belief updates from trial-wise PEs. To examine how participants scale PEs according to these CPP and RU when forming subsequent updates, we included the interaction of the absolute PE with the individual estimates of (1) CPP and (2) RU as effects (27, 28, 30). Thus, by referring to CPP- and RU-effects on belief updating, we mean the modulation of PE-driven learning by CPP and RU. Crucially, the regression β-weights of PE, PE^*^CPP, and PE^*^RU quantify to what extent a participant used the respective parameter to inform the update, whereas CPP and RU itself represent modulators of PE-driven belief updating. To evaluate the effect of each parameter at the group level, we tested the β-weights aggregated across participants against zero.

#### Association between PLE and Bayesian belief updating

To evaluate if the usage of PE, CPP, and RU differs as a function of PLE, we used multiple regression analyses. For each participant, we extracted the β-weights of each Bayesian model parameter (βPE, βCPP, and βRU), from the regression described in the previous section and used these to predict PLE. To elucidate how well participants adhered to optimal Bayesian learning overall, we correlated the variance explained by individual regression models on Bayesian updating (*R2*) with PLE.

#### Association between other self-report measures and Bayesian belief updating

To test for specificity of the relationship between PLE and altered belief updating, we repeated the regression analyses described in the previous paragraph and used the individual regression weights (βPE, βCPP, and βRU) to predict obsessive–compulsiveness (OCI-R), trait-anxiety (STAI-T), self-reported apathy (AES), alcohol use disorder (AUDIT), and the three self-report scores that constitute the PLE score (PDI, ASI, and CAPS). All self-report scores were normalized before being entered into the regression.

## Results

### Participants

The final sample included 300 participants (mean age = 25.63 ± 6.95 years) of whom 47.3% were identified as women (*n* = 142), 51.3% were identified as men, and 1.3% were identified as diverse (*n* = 4). The sample was international, with participants reporting an origin from 28 different countries (Supplementary Figure 1). Of all participants, 44% reported working full- or part-time, 43% being university students (overlap possible), and 18% reported being unemployed. Consumption of marihuana within the last month was reported by 14.3% (n=43) of the participants and consumption of amphetamines by one participant. Summary statistics of the psychiatric questionnaires are shown in Table 1 and distributions are displayed in Supplementary material.

**Table 1:** Descriptive statistics of self-reports.

**Self-report [Range]**	**Total sample Mean ±SD**
Psychotic-like experience score (z-transformed) [−1.31, 3.11]	0 ± .88
Peters delusion inventory [0.40]	7.43 ± 5.65
Aberrant salience inventory [0.29]	11.60 ± 7.06
Cardiff anomalous perception scale [0.32]	4.35 ± 4.45
Obsessive-compulsiveness inventory-revised [0.72]	18.88 ± 11.72
State-trait-anxiety inventory - trait [20.80]	47.46 ± 11.54
Apathy evaluation scale [18.72]	54.79 ± 7.95
Alcohol use disorder identification test [0.40]	5.09 ± 5

### Raw data analysis of observed behavior

**Figure 2:**
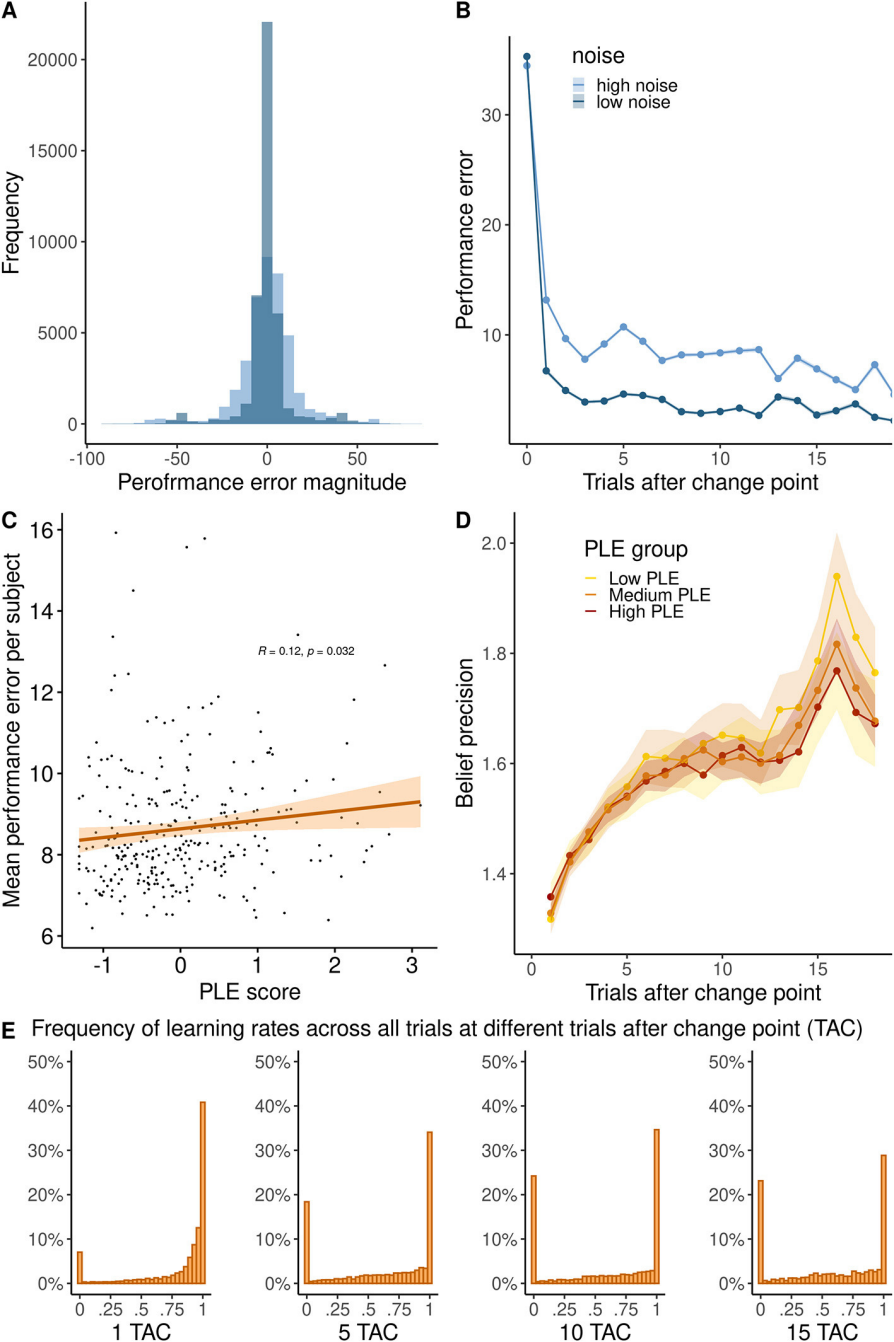
**(A)** Histogram of the performance error in high and low noise conditions. **(B)** Trajectory of performance error magnitude across trials after a change point. **(C)** Relationship between PLE and the average performance error per subject. **(D)** Precision of high and low noise runs across trials after change point in high (PLE 75% quartile), medium (PLE 25–75% quartile), and low PLE groups (25% quartile). **(E)** Histogram of learning rates (in percentage) showing the distribution at 1, 5, 10, or 15 trials after the change point (TAC).

The average trial-wise performance error (i.e., the deviation of individual predictions from the true mean) across subjects was *M* = 8.64 ± *SD* = 1.53 units (scale 0–100 units). Performance errors decreased after change points (the main effect of *TAC*: β = −0.3, *SE* = 0.009, 95% CI = [−0.32, −0.28], *p* < 0.001, Figure 2B), suggesting that participants made an effort to approximate the helicopter's locations after repeated observations. Reflecting that our noise manipulation increased the difficulty to track the helicopter's location, performance errors were higher in the high noise condition (main effect *noise*: β = −5.83 *SE* = 0.145, 95% CI = [−6.05, −5.62], *p* < 0.001, Figure 2A). Performance improved more across trials after a change point in the low noise compared to the high noise condition (interaction of *TAC* and *noise:* β = 0.13, *SE* = 0.012, 95% CI = [0.11, 0.16], *p* < 0.001). These results assure us that participants in our online study understood and performed the task as intended.

### Raw behavior in relation to psychotic-like experiences

To investigate our main research question, how PLEs were related to belief updating, we added individual PLE scores to the previous regression model. We found that participants with higher PLEs committed more performance errors overall (main effect of PLE: β = 0.26, *SE* = 0.11, 95% CI = [0.11, 0.16], *p* = 0.018, see Figure 2C). There were no significant interaction effects of PLE, TAC, and noise (full model reported in Supplementary material).

Next, we computed the theoretical precision of participant beliefs across trials after change points as a measure that reflects how many observations participants integrated to form their posterior beliefs. As shown in Figure 2E, participants mostly used learning rates of 0 or 1, limiting the overall precision. Precision significantly increased across trials after a change point (main effect *TAC*: β = 0.008, *SE* = .001, 95% CI [0.004, 0.008], *p* < 0.001, Figure 2D) and was higher when noise was low (main effect *noise*: β = −0.079, *SE* = 0.010, 95% CI [−0.071, −0.024], *p* < 0.001). Precision increased more across trials after a change point in the low compared to the high noise condition (interaction of *TAC* and *noise*: β = 0.025, SE = 0.001, 95% CI [0.019, 0.025], *p* < 0.001. Across trials after a change point, precision increased less in people with high PLE (interaction *TAC* and *PLE*: β = −0.003, SE = 0.0007, 95% CI [−0.004, −0.001], *p* < 0.001, Figure 2D), particularly in the low noise condition (interaction of *noise, TAC* and *PLE*: β = −0.016, SE = 0.006, 95% CI [−0.031–0.005], *p* = 0.012).

We found that PLEs were associated with slower learning (β = −0.03, *t*(298) = −3.326, *p* = 0.001), when participants observed large PEs (PE > 50), but not when participants observed small PEs (PE < 5) (β = −0.0006, *t*(298) = −0.046, *p* = 0.963). Repetition of this analysis with varying cutoffs is reported in the Supplementary material.

### Association between PLE and Bayesian belief updating

Averaged across all participants, the linear models predicting each participant's belief updates by PE, PE^*^CPP, and PE^*^RU accounted for 78% of the variance in participant's updates (*R*^2^ = 0.78 ± 0.14), and regression coefficients were all significantly different from zero (all *p* ≤ 0.001, Table 2), suggesting that the belief updating model captured participant's behavior well. As indicated by the regression coefficients (Table 2, participants updated their beliefs consistently according to PEs and adjusted the magnitude of their updates slightly according to CPP and RU, as indicated by small but positive coefficients for interaction terms. Overall, the variance explained by the computational model (*R2* of individual regressions) decreased with higher PLE (*r* = −0.17, *p* = 0.003).

**Table 2:** Regression coefficients (averaged across subjects) of PE, CPP, and RU predicting individual updates across high and low noise conditions.

	**Aggregated regression coefficients across subjects**		
	β**-weight [95% KI]**	***t*** **-value**	***P***
PE	0.77 [.76, 0.79]	90.52	< 0.001
PE: CPP	0.08 [.06, 0.10]	7.87	< 0.001
PE: RU	0.07 [0.04, 0.11]	4.77	< 0.001

**Figure 3:**
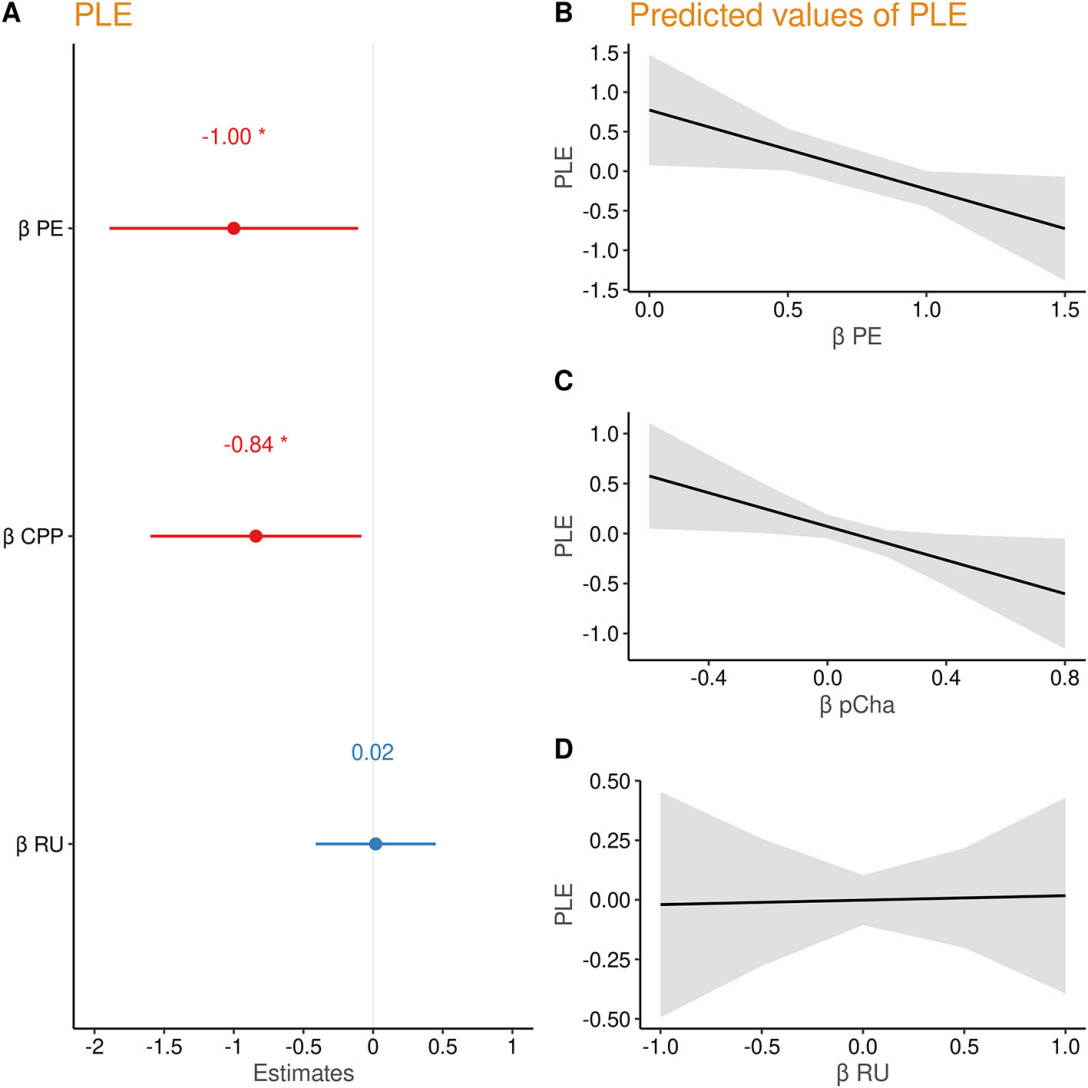
**(A)** Regression coefficients of prediction error (PE), change point probability (CPP), and relative uncertainty (RU) predicting PLE. **(B–D)** Marginal effects of each parameter on psychotic-like experiences (PLEs).

To examine whether belief updating according to PE and the model parameters (CPP and RU) were related to PLE, we entered the individual regression coefficients β_PE_, $\beta_{{\text{PE}}^{*}CPP}$, and $\beta_{{\text{PE}}^{*}RU}$ from the subject-wise regression into a second linear model on PLE score (PLE ~ β_PE_+$\beta_{{\text{PE}}^{*}CPP}$+$\beta_{{\text{PE}}^{*}RU}$). PLEs were predicted by lower β_PE_ (β = −1.00, *t* = −2.21, *p* = 0.028) and lower $\beta_{{\text{PE}}^{*}CPP}$ (β = −0.84, *t* = −2.19, *p* = 0.030), but not by $\beta_{{\text{PE}}^{*}RU}$ (β = 0.02, *t* = 0.09, *p* = 0.932) (see Figure 3). Thus, people with higher PLE updated beliefs less in response to prediction errors and failed to increase their updating after likely change points (additional analyses reported in Supplementary material).

### Association between other self-report measures and Bayesian belief updating psychiatric self-report measures

**Figure 4:**
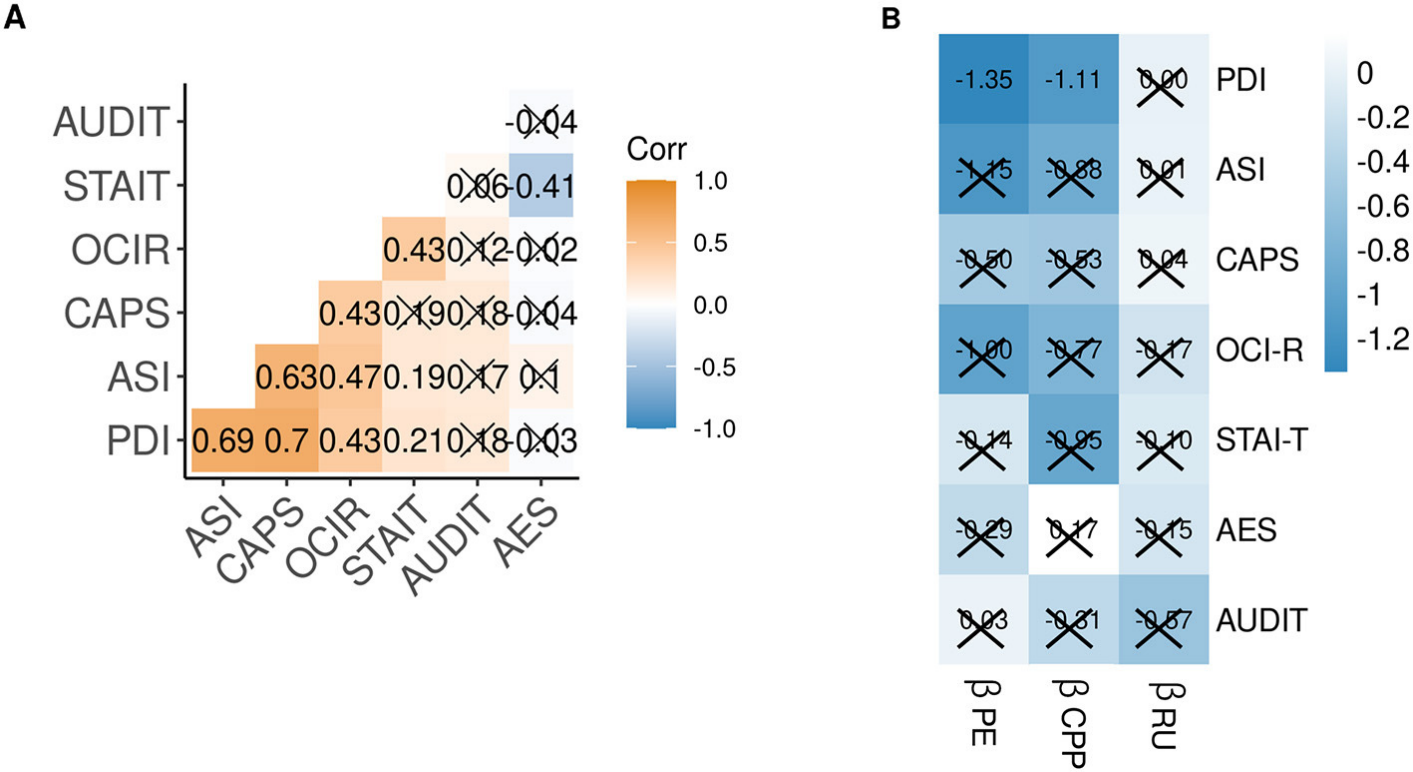
**(A)** Correlation matrix of z-scored self-reported psychiatric traits. **(B)** Matrix of regression weights predicting z-scored self-reported psychiatric trait scores from the individual Bayesian model-derived coefficients. Crossed cells *p* < 0.1, after *p*-value correction for multiple comparisons. PE, prediction error; CPP, change-point probability; RU, relative uncertainty; PDI, Peters Delusion Inventory; ASI, Aberrant Salience Inventory; CAPS, Cardiff Anomalous Perception Scale; OCI-R Obsessive Compulsiveness Inventory-Revised; STAI-T, State Trait Anxiety Inventory—Trait; AES, Apathy Evaluation Scale; AUDIT, Alcohol Use Disorder Identification Test.

In order to test the specificity of the relationship with PLE, we regressed the individual βPE, βCPP, and βRU on self-reported obsessive–compulsiveness (OCI-R), trait-anxiety (STAI-T), apathy (AES), alcohol use disorder (AUDIT), and the three self-report scores that constitute the PLE score (PDI, ASI, and CAPS). Intercorrelations of the self-report scores, and the relationship between individual βPE, βCPP, and βRU and self-reports are shown in Figure 4. Corroborating our results, only the relationship of PDI with βPE (*p* = 0.060) and with βCPP (*p* = 0.077) was trend-level significant after Benjamini–Hochberg correction for multiple comparisons. This suggests that people with high subclinical delusions update less according to PE magnitude and in response to likely change points. Without correction, these associations were significant (*p* < 0.05), as well as the relationship of ASI scores with βPE and βCPP. Limiting the specificity claims, we also observed a negative relationship of βCPP with trait anxiety and of βRU with alcohol use disorder scores (both only significant without *p*-value correction).

## Discussion

In order to gain a deeper understanding of the relationship between PLEs and altered belief updating, we conducted a behavioral online study among the general population. Participants had to update their beliefs about upcoming events based on noisy feedback in a task environment with sudden, unannounced change points. We found that PLEs were associated with overall less accurate beliefs, and a smaller increase in belief precision across observations after a change point has occurred. Participants with higher PLEs updated their beliefs less according to the observed prediction error (PE) and to likely change points.

The formation of beliefs about the world and oneself are critical in our conception of psychiatric disorders. They are also related to unspecific distress in non-psychiatric populations (43). Questioning the content and meta-cognition (e.g., truth claims, rationality, and function) of maladaptive beliefs is one of the central pillars of cognitive behavioral therapy (44). Therefore, a better understanding of how beliefs are developed and sustained may help us improve cognitive therapy and eventually mental wellbeing. This is particularly relevant for the hallmarks of PLEs, such as delusions, which are defined as false beliefs that are firmly sustained despite contrary evidence (45). Altered belief updating in dynamic environments is an established research finding in psychosis and can contribute to a mechanistic explanation for psychotic experiences (8, 10, 46). Yet, the exact inferential mechanisms to characterize these alterations remain to be clarified. Moreover, it is still unclear whether such alterations specifically underlie PLEs or are related to other dimensions of psychopathology.

First, in our belief updating paradigm, PLEs were associated with lower performance accuracy. Lower performance in probabilistic belief updating paradigms is an established finding in patients with schizophrenia (12, 13, 47) or first-episode psychosis (19) but also subclinical populations such as people with high paranoia (47–49), however not in people at a clinical high risk for psychosis (50, 51). Second, we observed that PLEs were associated with a smaller increase in belief precision across observations after a change point. This indicates that people with high PLEs integrated fewer previous observations to form their beliefs, although intuitively, sampling across more observations increases precision and consequently performance (16). Lower belief precision in the same task is also reported in patients with schizophrenia (16). Importantly, this measure of precision is based on behavioral data rather than the normative Bayesian estimate of precision. In particular, this measure uses the sequence of learning rates employed by the participant to infer how many prior observations are combined into the belief (52). In volatile environments, behavioral performance benefits not only from updating beliefs in responses to prediction errors but also from scaling the degree of updating according to the estimated belief uncertainty and the probability of environmental changes (53). This aligns with our findings that higher PLEs were not only associated with lower accuracy and precision but also with reduced belief updating and dynamic modulation of updating according to change point probability (CPP). In practice, this would drive slower learning after very large prediction errors, which often flag change points. Increasing the learning rate after an environmental change is crucial to facilitate disregarding an obsolete representation of the world and acquiring a new belief. This is in line with the conceptualization of delusions as rigid beliefs that resist contrary evidence, since beliefs are adjusted slower despite large prediction errors, which reflect contrary evidence in our task.

From a computational perspective, the slower updating of beliefs following environmental change points suggests a strong prior belief that is resitant to modification. Although in prior research, strong (perceptual) priors and a bias toward top–down information were mostly related to hallucinations (54, 55), it is possible that similar mechanisms in hallucinations and delusions are at play (46). As such, plenty of research studies support the relationship between delusions and a jumping-to-conclusion bias, which reflects the fast acquisition of a strong prior (if no belief existed before). A previous study, using a variant of the beads task (15), refined this by showing that delusions were specifically related to overweighting of initial evidence early in the learning process, and to a failure to adequately integrate later information. A meta-analysis on evidence integration biases supports this further (56). They found that psychosis was not only related to jumping-to-conclusion bias but also to a bias against disconfirmatory evidence, suggesting reduced integration of conflicting evidence into this prior. This resembles our findings that PLEs were associated with slower updating behavior in response to large prediction errors. In future studies, it would be interesting to specifically investigate whether reduced belief acquisition is equally altered in PLEs when a novel belief must be established or when an already established belief must be overridden, by integrating contradictory evidence in favor of a new belief.

Although not the main goal of our task, we tried to tap into the aberrant salience construct (57, 58). The aberrant salience hypothesis poses that anomalous percepts or beliefs result from neutral events, such as noise, that are erroneously interpreted as salient or meaningful (8, 57). We found some evidence for an aberrant salience phenomenon in higher PLEs since PLEs were related to less adherence to belief updating to the quasi-optimal Bayesian computational model, resembling a more noisy response process.

We assessed other psychiatric traits to test for the specificity of our results and examined the subcomponents of the PLE score (Peters Delusion Inventory [PDI], Aberrant Salience Inventory [ASI], and Cardiff Anomalous Perceptions Scale [CAPS]). We observed a trend-wise negative relationship between updating adherence to PE magnitude and unexpected environmental changes (CPP) with self-reported subclinical delusions (PDI). We observed no relationship with any other psychiatric trait. In line with this, the previous study using a similar task did not find a relationship between updating behavior and the transdiagnostic factors “anxious depression,” “social withdrawal,” or “compulsive behavior and intrusive thought” either (30), suggesting that altered belief updating is specifically related to psychosis.

### Limitations and implications for future work

The present study has several limitations. First, the reported effects are rather small. Yet, our main analyses were preregistered, lending trust in the *a priori* hypotheses, and replication of these results is needed to increase confidence in our findings. Ideally, future studies could adopt larger sample sizes and as suggested by one reviewer, adopt a strategy to increase the sample size until a point where the evidence for findings exceeds a pre-defined Bayes Factor. Unfortunately, the online administration limited our ability to control possible distractions from the task and the self-assessment, and we did not acquire neurocognitive measures. Indeed, cognitive performance is often reported to account for altered belief updating in psychosis (10, 16), although our sample was not clinical but had rather subclinical symptoms. In our paradigm, the mouse on each new trial is located at the position of the previous trial. While this prevents confounds due to working memory demands, it may lead to confounds due to motion or perseverance, as pointed out by a reviewer. Therefore, the mouse could on every new trial be located at a random position on the scale. Prior work has shown that this prevents perseverance on previous mouse locations, but does not result in qualitative changes in normative learning and may even introduce a random bias (41). Yet, these potential confounds should be taken into consideration for future task designs. We strongly encourage longitudinal designs for future work on belief updating in psychosis that would allow investigating if certain metrics of belief updating are associated with subsequent increase or decrease of PLEs or progression of subclinical PLEs to clinically relevant psychotic symptoms. This could be achieved by investigating groups at high risk for psychosis, such as first-degree relatives or individuals with subclinical psychotic experiences. Not only groups at high risk for psychosis could be of interest but also those at high risk for other psychiatric traits. We would like to highlight the great potential of online studies to collect data from multinational samples. In psychological research, western, industrialized, and rich nationalities are often considered representational, as suggested by the fact that the nationality of these samples, as compared to minorities, is less often mentioned in the title of a paper (59, 60). Although the present sample is of course not representative of the world's population, a great number of participants originate from South Africa and Mexico. Conducting online studies may aid in reaching out to these populations and eventually increase their representation in research.

## Conclusion

In summary, the present study highlights that altered belief updating is related to psychotic-like experiences (PLEs) in subclinical populations. Our results support the notion that people with high PLEs show a lower belief accuracy and update less in response to prediction errors and likely change points. They seemed to stick with an established prior belief and updated this belief slower when faced with contradictory evidence. Slower belief updating and less accurate predictions about future states of the environment may be involved in the formation of delusion-like rigid beliefs.

## Data availability statement

The datasets presented in this study can be found in online repositories. The names of the repository/repositories and accession number(s) can be found below: https://github.com/SophieFromm/HeRoine.git.

## Ethics statement

The studies involving human participants were reviewed and approved by Ethics Committee of the Charité -Universitätsmedizin Berlin. The patients/participants provided their written informed consent to participate in this study.

## Author contributions

SF, LW, TK, and FS contributed to the study conception and design. SF and AK performed the data collection. SF, LW, and AK conducted the data analysis under the supervision of FS and MN. SF wrote the task code and the first draft of the manuscript. All authors commented on previous versions of the manuscript and read and approved the final manuscript.
