# Supplementary material for: Computational mechanisms of belief updating in relation to psychotic-like experiences
Source: Front Psychiatry. 2023 May 5;14:1170168. doi: 10.3389/fpsyt.2023.1170168 (PMC10196365; doi:10.3389/fpsyt.2023.1170168)
Supplement: Supplementary file 1 [file Data_Sheet_1.docx]

*Supplementary Materials*

**Computational mechanisms of belief updating
in relation to psychotic-like experiences**

*****[**Sophie Pauline Fromm**](https://osf.io/4ywpz)**, Lara Wieland, Arne Klettke, Mathew Nassar, Teresa Katthagen, Sebastian Markett, Andreas Heinz and Florian Schlagenhauf**

* Correspondence Sophie Fromm
sophie.fromm@charite.de

[**Sample Characteristics** 2](#_Toc131414668)

[**Categorizing single trial learning rates** 2](#_Toc131414669)

[**Computation of precision** 2](#_Toc131414670)

[**Linear mixed regression of performance error and precision** 4](#_Toc131414671)

[**Association of PLE with learning rate dynamics across prediction errors** 6](#_Toc131414672)

[**Binned regression of prediction errors across trials after change points** 8](#_Toc131414673)

[**Regression of PLE on model parameters on trials with large PEs** 9](#_Toc131414674)

[**Regression of sums of PDI and CAPS subscales on model parameters** 9](#_Toc131414675)

[**Regression of PLE on model parameters separate in low and high noise trials** 10](#_Toc131414676)

[**Relationship between obsessive-compulsiveness and belief updating** 11](#_Toc131414677)

[**Mixed Regression on model parameters** 11](#_Toc131414678)

[**Factor analysis on measures of PLE** 12](#_Toc131414679)

[**Results of exploratory factor analysis of PLE** 21](#_Toc131414680)

[**Histogram of model parameters** 24](#_Toc131414681)

## **Sample Characteristics**


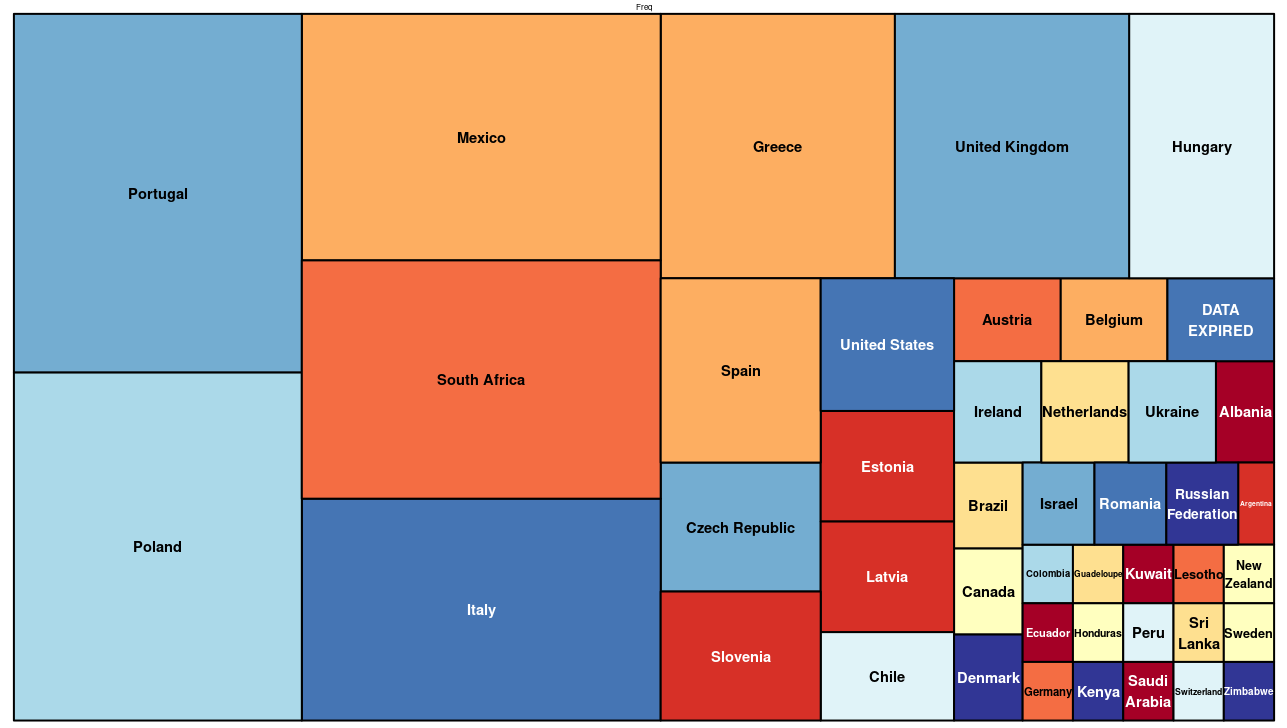


**Supplementary Figure 1**. Relative country of origin of the total sample.

## **Categorizing single trial learning rates**

Learning rates were categorized into non- (α ≤.1), moderate (.1 < α ≤ .9) and total updates (α >.9) to quantify the degree of updating. Missed trials as well as the last trial of each run were not counted since participants did not update their beliefs on that last trial and no learning rate could be computed. Across all participants, the degree of updating differed significantly between noise conditions (χ2 = 595.95, p <.001). In the low noise condition, we observed more non-updates (8179 vs. 10632), less moderate (14650 vs. 13762) and less total updates (17943 vs. 16399) than in the high noise condition. We computed the fraction of updates from non-, moderate and total learning for each subject and correlated each fraction with PLE. None of these correlations was significant (all p-values > .376).

## **Computation of precision**

We computed the precision according to Nassar et al. (Brain, 2021). For each trial, we computed a weight matrix, with learning rates of each trial in the diagonal of that matrix (for example trial 5 with α = 0.9 in the example matrix below). Cells on the left side of each trial in the matrix comprised the weights of the previous trials. These weights sum up to 1- α (in our example = 0.1) and represent the fraction of the current belief that is informed by previous beliefs. The share that each previous trial had of 1- alpha was proportional to the learning rate on that previous trial (in our example the two previous trials share equal proportions = .05). Thereby, we assume that the belief on each trial is formed by integrating previous observations weighted by the respective learning rate. Crucially, previous outcomes should gain no weight immediately after a change point, which is achieved by a learning rate α = 1, like in trial 3 or 10. As soon as a learning rate equal to one occurs, previous trials gain no weight (1-1 = 0). The precision was then computed for each trial according to formula 1 and 2.

Example weight matrix of lagged weights:

|  | 1 | 2 | 3 | 4 | 5 | 6 | 7 | 8 | 9 | 10 | 11 | 12 |
| --- | --- | --- | --- | --- | --- | --- | --- | --- | --- | --- | --- | --- |
| 1 | 0,00 | - | - | - | - | - | - | - | - | - | - | - |
| 2 | 0,23 | 0,68 | - | - | - | - | - | - | - | - | - | - |
| 3 | 0,00 | 0,00 | 1,00 | - | - | - | - | - | - | - | - | - |
| 4 | 0,00 | 0,00 | 0,48 | 0,52 | - | - | - | - | - | - | - | - |
| **5** | **0,00** | **0,00** | **0,05** | **0,05** | **0,90** | - | - | - | - | - | - | - |
| 6 | 0,00 | 0,00 | 0,01 | 0,01 | 0,12 | 0,87 | - | - | - | - | - | - |
| 7 | 0,00 | 0,00 | 0,00 | 0,00 | 0,06 | 0,44 | 0,49 | - | - | - | - | - |
| 8 | 0,00 | 0,00 | 0,00 | 0,00 | 0,06 | 0,44 | 0,49 | 0,00 | - | - | - | - |
| 9 | 0,00 | 0,00 | 0,00 | 0,00 | 0,01 | 0,11 | 0,12 | 0,00 | 0,75 | - | - | - |
| 10 | 0,00 | 0,00 | 0,00 | 0,00 | 0,00 | 0,00 | 0,00 | 0,00 | 0,00 | 1,00 | - | - |
| 12 | 0,00 | 0,00 | 0,00 | 0,00 | 0,00 | 0,00 | 0,00 | 0,00 | 0,00 | 0,05 | - | - |
| 13 | 0,00 | 0,00 | 0,00 | 0,00 | 0,00 | 0,00 | 0,00 | 0,00 | 0,00 | 0,00 | 0,00 | 1,00 |

Formula 1)


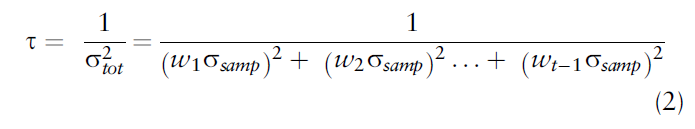


Formula 2)

Precision =
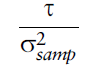


For the regression analysis, we excluded the very first block of each run (all trials until the first change point occurred). In the initial trials, participants were likely to explore the functioning of the mouse and task. Since each trial influences the precision of the following trials until a change point (or learning rate of 1) occurs, we had to exclude these initial trials to prevent confounding. Also trials later than 17 trials after the most recent change point were excluded, since only five blocks exceeded a length of 17 trials.

## **Linear mixed regression of performance error and precision**

We investigated how performance errors evolved after change points in the low and high noise condition using a linear mixed regression model and how these dynamics associate with PLE. For this analysis, we excluded trials when a change point occurred as well as trials that occurred later than 17 trials after the most recent change point, since only five blocks exceeded a length of 17 trials. This resulted in a total of 70600 observations. We then compared the full model to all reduced model versions and evaluate the best fitting model using the buildmer function from the buildmer R-toolbox. As the default criterion is the likelihood-ratio test (LRT) based on chi-square mixtures to test for the contribution of terms to model fit. Additionally, we report model selection based on the Akaike Information Criterion (AIC). The full model comprised a main effect of *trials after change point* (*TAC*), *noise* and *PLE*. All terms were allowed to interact with each other. Additionally, we specified a random intercept for *subjects* and random slopes for *TAC*, *noise* and the interaction of both terms.

Performance error ~ (TAC * noise * *PLE*) + (1 + TAC * noise | subject)

Results: According to both LRT and AIC, the best model comprised a main effect of *noise*, *TAC*, *PLE* and an interaction of noise and *TAC, as* well as a random effect for subjects (Supplementary Table 1). Models that featured random slopes did not converge.

**Supplementary Table 1**. Linear mixed model predicting performance error by *noise, trials after change points (TAC)* and *PLE*.

| Effect | Estimate | *SE* | *t-value* | 95% CI | *p-value* |
| --- | --- | --- | --- | --- | --- |
| Fixed effects |  |  |  |  |  |
| Intercept | 11.27 | 0.12 | 92.21 | [11.03, 11.51] | < .001 |
| noise | -5.84 | 0.11 | -52.94 | [-6.05, -5.62] | < .001 |
| TAC | -0.30 | 0.01 | -34.53 | [-.32, -.28] | < .001 |
| PLE (centered) | 0.26 | 0.11 | 2.36 | [.04, .48] | .018 |
| noise * TAC | 0.13 | 0.01 | 10.90 | [.11, .16] | < .001 |
| Random effects |  |  |  |  |  |
| Subject intercept | Variance 2.666 | Std 1.633 |  |  |  |

Note. Number of observations: 70600, Number of subjects: 300

In the same manner, we compared models regarding the precision measure with the following full model.

Precision ~ (TAC * noise * *PLE*) + (1 + TAC * noise | subject)

According to LRT, the best model comprised main effects of noise, TAC and PLE and 2-way and 3-way interactions as reported in the manuscript. According to AIC, all main-effects and 2-way interactions were included in the best model, as reported below.

**Supplementary Table 2**. Linear mixed model predicting precision by *noise, trials after change points (TAC)* and *PLE*.

| Effect | Estimate | *SE* | *t-value* | 95% CI | *p-value* |
| --- | --- | --- | --- | --- | --- |
| Fixed effects |  |  |  |  |  |
| Intercept | 1.45 | 0.021 | 70.8 | [1.41, 1.49] | < .001 |
| noise | -0.079 | <.001 | 8.38 | [0.006, 0.009] | < .001 |
| TAC | 0.008 | 0.010 | -7.59 | [-0.099, -0.058] | < .001 |
| PLE (centered) | -.014 | 0.023 | 0.61 | [-0.040, 0.051] | .541 |
| noise * TAC | -.025 | 0.001 | 20.36 | [-0.004, <0.001] | < .001 |
| TAC * PLE | -.003 | <.001 | -4.6 | [-0.023, 0.023] | < .001 |
| Noise * PLE | -.016 | .006 | -2.52 | [-0.005, <0.001] | .012 |
| Random effects |  |  |  |  |  |
| Subject intercept | Variance 0.11 | Std 0.33 |  |  |  |

*Note. Number of observations: 65861, Number of subjects: 300*

## **Association of PLE with learning rate dynamics across prediction errors**

As reported in the manuscript, we computed regressions predicting learning rates by PLE of trials with specific PE-magnitude. This allowed us to examine whether associations of PLE and learning rate dynamics are more pronounced for small or large PEs. Our results suggest that participants with low PLE learned faster from large PEs with a magnitude > 20 (Supplementary Table 2). Supplementary Figure 2 shows learning rate dynamics across PE-magnitude with (A) showing the whole PE-range and (B) showing only trials on which small PEs were observed (PE < 10) for different groups of subjects. Groups were split according to PLE - scores. Supplementary Figure 2 (B) suggests that high PLE was associated with faster learning for small PEs. However, this did not present as a significant result in the here reported analysis.

**Supplementary Table 3**. Regression predicting learning rates for trials on which participants observed specific magnitudes of absolute prediction errors.

| PE - magnitude | β PLE | *t-value* | *p-value* |
| --- | --- | --- | --- |
| < 1 | 0.002 | 0.18 | 0.854 |
| < 2 | < 0.001 | 0.01 | 0.995 |
| < 3 | 0.003 | 0.22 | 0.838 |
| < 4 | 0.001 | 0.09 | 0.929 |
| < 5 | <0.001 | 0.05 | 0.963 |
| > 10 | -0.013 | -1.81 | 0.071 |
| > 20 | -0.018 | -2.47 | **0.014** |
| > 30 | -0.021 | -2.64 | **0.009** |
| > 40 | -0.022 | -2.67 | **0.008** |
| > 50 | -0.029 | -3.26 | **0.001** |


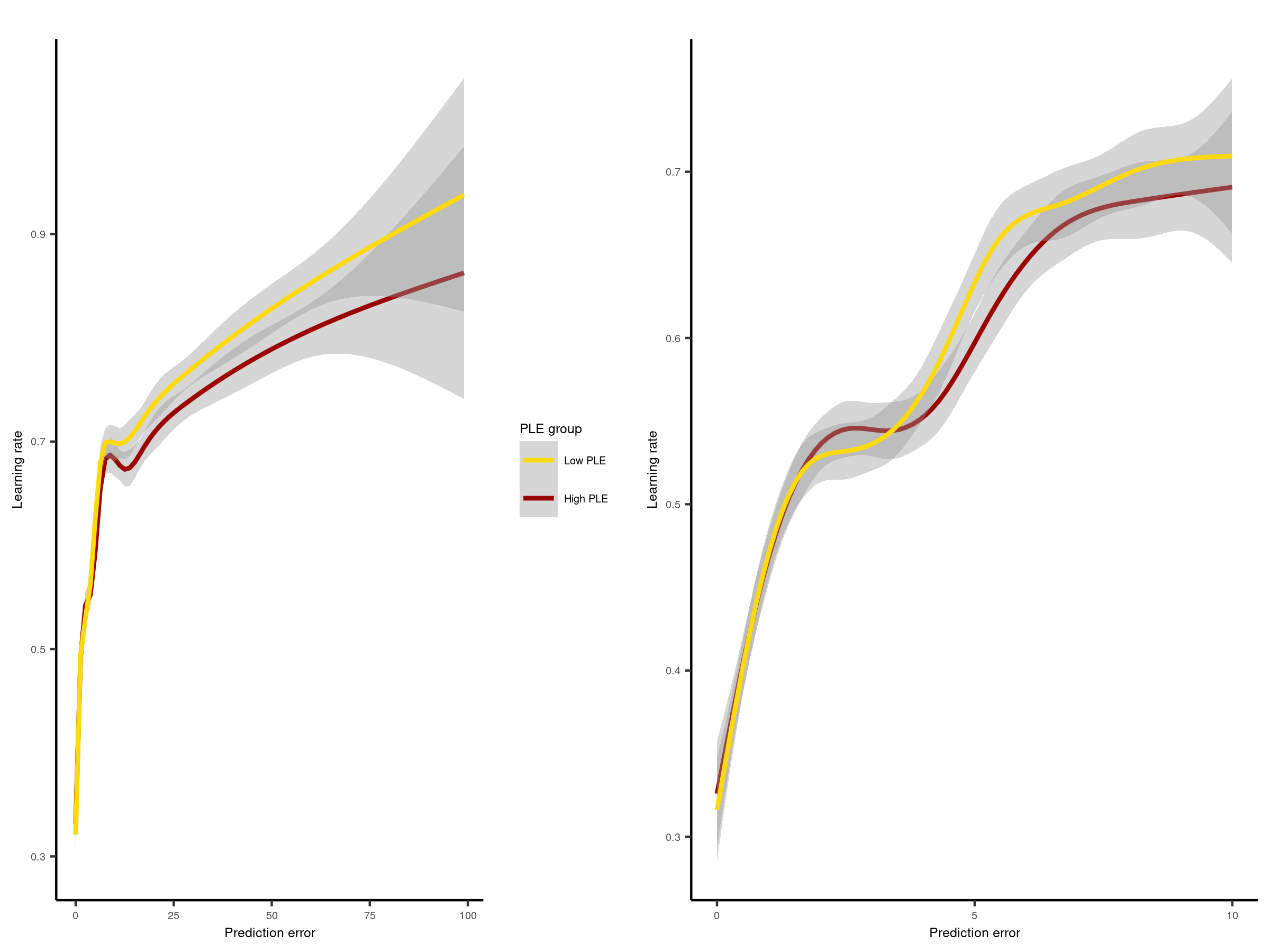


**Supplementary Figure 2.** A) Learning rate smoothed across prediction errors for groups split by quartiles of PLE-magnitude (25%, 75%) for PE magnitude of the full range (0-100) and for a window of small PEs (0-10).

## **Binned regression of prediction errors across trials after change points**

We followed up our finding that PLE was negatively associated with the PE-coefficients using a binned regression-analysis. We binned the data according to trials after change points (TAC) for each subject and computed individual regressions predicting the update by the PE within each bin. Examining the regression coefficients of PE across TAC allows us to investigate to what extent participants updates adhered to the PE-magnitude at different levels of uncertainty. As expected, we saw that participants with high PLE showed lower PE-coefficients for early TAC as compared to low PLE. PLE were negatively correlated with PE-coefficients across all TAC (*r* = -.038, *p* =.01) and more strongly at early TACs, such as TAC < 4 (*r* =-.11, *p* <.001).


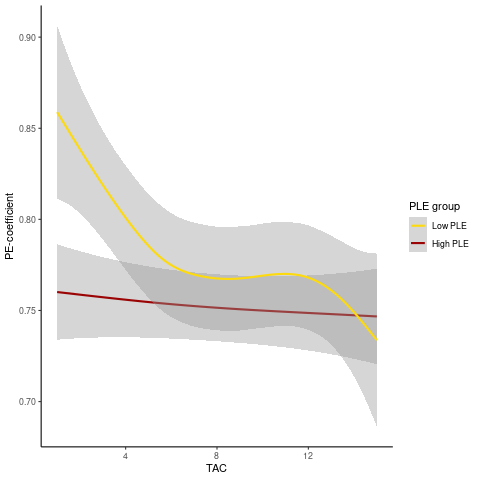


**Supplementary Figure 3.** A) Coefficients predicting updates by PE across different trials after change point (TAC) for groups split by quartiles of PLE-magnitude (25%, and 75%). Shaded area shows the confidence interval.

## **Regression of PLE on model parameters on trials with large PEs**

As a reviewer pointed out, movement artifacts could confound computations of LRs from small prediction errors more easily. We excluded trials with small PEs (absolute PE < 5) from the model based regression analysis across all trials. We then repeated our main analysis, predicting updates by PE, PE*CPP and PE*RU. We could confirm the findings reported in the manuscript. Again, PLE was associated with smaller PE-coefficients (β = -.97, t = -2.10, *p* = .037) and smaller PE*CPP coefficients (β = -.73, t = -2.07, *p* = .040), whereas PE*RU was no significant predictor of PLE (β = .07, t = 0.35, *p* = .730). Thus, also in trials with only large PEs, people with high PLE updated less according to PEs and CPP.

## **Regression of sums of PDI and CAPS subscales on model parameters**

As pointed out by a reviewer, the PDI subscales (distress, preoccupation, conviction) and CAPS subscales (distress, intrusiveness, and frequency of anomalous experience) are often adopted and may provide a clinically more relevant measure as compared the mere occurrence of the experience described in each item. As such, we computed sumscores of the subscales for each measure and repeated the regression analysis of model parameters on PLE, with the subscale sumscores.

**Supplementary Table 4**. Linear mixed model predicting scores of the CAPS- and PDI-subscale by model parameters.

|  | | **PDI-subscale scores** | | | **CAPS- subscale scores** | |
| --- | --- | --- | --- | --- | --- | --- |
| Effect | Estimate  [95% CI] | | *p-value* | Estimate  [95% CI] | | *p-value* |
| Intercept | 262.67 | | **<.001** | 50.29 | | **.006** |
| PE | -156.84 | | **.007** | -15.53 | | .477 |
| PE*CPP | -133.09 | | **.007** | -29.38 | | .113 |
| PE*RU | -4.56 | | .871 | 2.56 | | .830 |

## **Regression of PLE on model parameters separate in low and high noise trials**

To investigate the effect of environmental noise on belief updating, we ran the regression models described in H3 separately for each noise condition, compared coefficients using t-tests and again probed their relationship with PLE. Regression coefficients of PE and CPP in low versus high noise conditions differed significantly as reported in Table 2. The impact of PE and CPP on updating was higher in the low noise condition, whereas updating adhered more to the RU trajectory in the high noise condition. We specified a second-level linear regression model of coefficients on PLE for low and high noise runs separately. The relationship of the βCPP and βCPP with PLE was only significant in the low noise condition, suggesting that alterations in belief updating related to PLE are less pronounced when environmental noise is high. Lastly, we computed the difference between coefficients from high and low noise regressions and regressed the difference scores on PLE. As shown in Supplementary Table S, none of these coefficients were significant, suggesting that the differentiation in use of PE, CPP and RU between high and low noise runs is not associated with PLE.

**Supplementary Table 5.** Regression coefficients of high and low noise runs separately.

|  | **Low noise** | **β on PLE** | **High noise** | **β on PLE** | **Difference low vs. high noise** | **β on PLE** |
| --- | --- | --- | --- | --- | --- | --- |
|  | Estimate [95% CI] | Estimate *(p)* | Estimate [95% CI] | Estimate *(p)* | *t-value (p)* | Estimate *(p)* |
| **PE** | .80 [.77, .82] | -1.08 (.012)* | .77 [.75, .79] | -.676 (.101) | 2.62 (.009)* | -.218 (.618) |
| **PE*CPP** | .11 [.08,.14] | -.91 (.007)* | .07 [.05, .09] | -.465 (.137) | 2.45 (.015)* | -.181 (.526) |
| **PE*RU** | .07 [.03, .12] | .16 (.343) | .12 [.08, .15] | -.166 (.34) | -1.67 (.095) | -.132 (.278) |

## **Relationship between obsessive-compulsiveness and belief updating**

## Prior studies suggest altered belief updating in obsessive compulsive disorder (Marzuki et al., 2022) and a particular deficit in using metacognitive information, such as the confidence about beliefs to inform updating actions (Vaghi et al., 2017). We used the revised obsessive-compulsiveness inventory (OCI-R, Foa et al., 2002) to assess subclinical self-reported obsessive-compulsiveness. Previous research using a similar task suggested that subclinical obsessive-compulsiveness also relates to impaired action-confidence coupling, resembling a reduction of explicit confidence in beliefs to inform updating actions. Yet, the updating behavior itself according to CPP and RU was unaffected by subclinical obsessive-compulsiveness (Seow and Gillan, 2020). In our study, OCI-R scores were highly correlated with PLE measures (r = .43 - .47, p <.001, Figure 4 in the manuscript). Similar to associations with PLE, model parameters βPE and βPE*CPP predicted OCI-R scores trend-wise (βPE = -1.00, p = .052; βPE*CPP= -.77, p = .079), while βPE*RU was no significant predictor (β PE*RU = -.17, p = .485). When correcting for multiple testing for the number of selfreport questionnaires, only the association with PDI remained significant. Not only in our study, but also in other reports an overlap between paranoid ideation, predisposition to hallucinations with obsessive-compulsiveness and metacognitive beliefs in non-clinical samples was shown (Hagen, K., Solem, S., Opstad, H.B. et al., 2017). While we cannot rule out that our study was underpowered to detect relationships with obsessive-compulsiveness reliably, our results suggest a more pronounced relationship of updating alterations with PLE.

## **Mixed Regression on model parameters**

To follow up the two-level approach, we set up a mixed effects model and explored random effects via model comparisons in terms of convergence and fit to allow drawing conclusions on the population level. The full model comprised the same fixed effects as in the previous regression 1) PE 2) PE*CPP and 3) PE*RU. Each coefficient was allowed to interact with the centered PLE-score, thereby testing if the respective parameter was used to a different degree depending on the PLE score. Using the buildmer function in R, we specified a full model as follows:

Update ~ (PE + PE * CPP + PE * RU)*(PLE_cent) + (1 + X1 * X1X2 * X1X3 | Subject)

The best model, comprised main effects of PE, CPP and RU as well as an interaction of PLE with PE. The model did not include a random intercept or slope and is reported in Supplementary Table 4.

**Supplementary Table 6.** Best model predicting belief updates by Bayesian model parameters and PLE

| Effect | Estimate | SE | *t-value* | 95% CI | *p-value* |
| --- | --- | --- | --- | --- | --- |
| Fixed effects |  |  |  |  |  |
| Intercept | .071 | .028 | 2.5 | [.015, .013] | <.0125 |
| PE | .784 | .003 | 246.92 | [.777,.79] | <.001 |
| PE*CPP | .042 | .005 | 9.26 | [.033,051] | <.001 |
| PE*RU | .029 | .008 | 3.62 | [.0134, .045] | <.001 |
| PLE | -.033 | .032 | -1.04 | [-.096, .03] | .301 |
| PE*PLE | -.016 | .002 | -8.26 | [-.02, -.012] | <.001 |

 Note. Number of observations: 28195, Number of subjects, 300

**Factor analysis on measures of PLE**

We used exploratory factor analysis to determine the underlying factor structure of the PLE-questionnaires (PDI, CAPS, ASI). Whereas the PDI was designed to measure delusional ideation in the general population (40 items, Cronbach’s α = .88) (24), CAPS was designed to measure perceptual anomalies (32 items, Cronbach’s α = .87) (25). Both these questionnaires ask whether participants have had a certain experience and upon confirmation require participants to rate distress, preoccupation, and conviction (PDI) or distress, intrusiveness and frequency (CAPS) of that experience. We only used the ratings of whether participants have had the respective experience and disregarded the follow-up questions to achieve a consistent response format as required for factor analyses. The ASI comprises 30 items (Cronbach’s α = .89) in a binary response format and asks participants to rate experiences related to aberrant salience, meaning the incorrect assignment of salience, significance, or importance to otherwise innocuous stimuli (26). Since the questionnaires overlap to some extent, our goal was to use all information and still derive underlying dimensions that are as distinct as possible.

We applied Maximum Likelihood –Exploratory factor analysis (ML-EFA) to extract the factors and applied different methods to determine the number of factors. These were Kaiser-Guttman Criterion, Scree-Test, EKC criterion, Parallel analysis, Minimum Average partial test (MAP-Test). We applied oblique oblimin factor rotation that assumes correlated factors. Factors and all items are reported in the supplementary material and were interpreted in a multi-professional team of one psychiatrist and three psychologists. After completion of the interpretation, the derived factors were regressed on the individual coefficients βPE, βCPP and βRU that we derived from the Bayesian normative model.

**Factor loadings of single items**

**Supplementary Table 7**. Factor loadings of a Maximum Likelihood Analysis using oblimin rotation.

| Item | Factor | | | | | |
| --- | --- | --- | --- | --- | --- | --- |
|  | Aberrant salience | Delusional ideations and auditory alterations | Tactile, gustatory and olfactory alterations | Religious and grandiose beliefs | Distorted body experience and external control | h^2^ |
| asi_11 | 0.77 |  |  |  | -0.30 | 0.61 |
| asi_6 | 0.76 |  |  |  |  | 0.74 |
| asi_29 | 0.75 |  |  |  |  | 0.69 |
| asi_2 | 0.70 |  |  |  |  | 0.63 |
| asi_17 | 0.69 |  |  |  |  | 0.66 |
| asi_28 | 0.65 |  |  |  |  | 0.64 |
| asi_12 | 0.62 |  |  |  |  | 0.54 |
| asi_5 | 0.61 |  |  |  |  | 0.56 |
| asi_22 | 0.60 |  | 0.34 |  |  | 0.61 |
| asi_3 | 0.57 |  |  |  |  | 0.49 |
| asi_16 | 0.57 |  |  |  |  | 0.55 |
| asi_10 | 0.55 |  |  |  |  | 0.52 |
| asi_27 | 0.55 |  |  |  |  | 0.50 |
| asi_9 | 0.50 |  |  |  |  | 0.48 |
| asi_14 | 0.46 | 0.31 |  |  |  | 0.59 |
| asi_24 | 0.45 |  | -0.33 |  |  | 0.36 |
| asi_23 | 0.42 |  |  |  |  | 0.47 |
| asi_4 | 0.41 |  |  | 0.30 |  | 0.41 |
| asi_25 | 0.39 |  |  |  |  | 0.48 |
| asi_1 | 0.38 |  |  |  |  | 0.46 |
| asi_26 | 0.36 |  |  |  |  | 0.45 |
| asi_15 | 0.33 |  |  |  |  | 0.40 |
| asi_21 | 0.32 |  |  |  |  | 0.29 |
| asi_18 | 0.31 |  |  |  |  | 0.37 |
| caps_11 |  | 0.62 |  |  |  | 0.67 |
| pdi_15 |  | 0.59 |  |  |  | 0.56 |
| pdi_5 |  | 0.54 |  |  |  | 0.40 |
| caps_7 |  | 0.53 |  |  |  | 0.53 |
| pdi_10 |  | 0.53 |  |  |  | 0.52 |
| caps_3 |  | 0.51 |  |  |  | 0.51 |
| pdi_37 |  | 0.51 |  |  |  | 0.51 |
| asi_19 |  | 0.44 |  | 0.32 |  | 0.54 |
| pdi_12 |  | 0.43 |  |  |  | 0.47 |
| caps_6 |  | 0.43 |  |  |  | 0.38 |
| pdi_7 |  | 0.43 |  |  |  | 0.26 |
| pdi_36 |  | 0.42 |  |  |  | 0.44 |
| pdi_16 |  | 0.42 |  |  |  | 0.45 |
| caps_27 |  | 0.40 |  |  |  | 0.36 |
| caps_17 |  | 0.39 |  |  | 0.33 | 0.39 |
| pdi_38 |  | 0.37 | 0.36 |  |  | 0.58 |
| caps_32 |  | 0.37 | 0.33 |  |  | 0.37 |
| pdi_30 |  | 0.33 |  |  |  | 0.28 |
| pdi_35 |  | 0.33 |  |  |  | 0.26 |
| pdi_22 |  | 0.33 | 0.31 |  |  | 0.44 |
| caps_18 |  |  | 0.89 |  |  | 0.76 |
| caps_25 |  |  | 0.69 |  |  | 0.65 |
| caps_29 |  |  | 0.68 |  |  | 0.57 |
| caps_30 |  |  | 0.59 |  | 0.38 | 0.67 |
| caps_15 |  | 0.34 | 0.48 |  |  | 0.53 |
| pdi_28 |  |  | 0.47 |  |  | 0.48 |
| caps_21 |  |  | 0.45 |  | 0.33 | 0.60 |
| caps_8 |  |  | 0.45 |  |  | 0.34 |
| pdi_32 |  |  | 0.42 |  |  | 0.38 |
| caps_23 |  |  | 0.41 |  |  | 0.50 |
| caps_22 |  |  | 0.36 |  | 0.31 | 0.49 |
| caps_1 | 0.33 |  | 0.36 |  |  | 0.45 |
| pdi_9 |  |  | 0.33 | 0.30 |  | 0.55 |
| caps_5 |  |  | 0.33 |  |  | 0.32 |
| caps_20 |  |  | 0.32 |  |  | 0.29 |
| pdi_8 |  |  | 0.32 |  |  | 0.32 |
| pdi_21 |  |  |  | 0.89 |  | 0.93 |
| pdi_25 |  |  |  | 0.89 |  | 0.89 |
| pdi_18 |  |  |  | 0.59 |  | 0.50 |
| pdi_17 |  |  |  | 0.51 |  | 0.48 |
| pdi_31 |  |  |  | 0.48 |  | 0.44 |
| pdi_19 |  |  |  | 0.47 |  | 0.40 |
| pdi_1 |  |  |  | 0.44 |  | 0.53 |
| asi_7 |  |  |  | 0.44 |  | 0.55 |
| pdi_26 |  | 0.30 |  | 0.41 |  | 0.53 |
| asi_13 | 0.30 |  |  | 0.39 |  | 0.61 |
| caps_9 |  |  |  |  | 0.63 | 0.65 |
| pdi_29 |  |  |  |  | 0.61 | 0.58 |
| pdi_39 |  |  |  | 0.30 | 0.50 | 0.70 |
| pdi_4 |  |  |  |  | 0.49 | 0.49 |
| caps_14 |  |  |  |  | 0.44 | 0.34 |
| pdi_11 |  | 0.38 | 0.30 |  | 0.38 | 0.54 |
| pdi_23 |  |  |  |  | 0.31 | 0.23 |

Note. loadings under .3 are not displayed; loadings of excluded factors 6 and 7 are not shown;

**Supplementary Table 8**. Item abbreviation with associated item statements for PDI, CAPS, ASI.

| Item Abbreviation | Item statement |
| --- | --- |
| pdi_1 | Do you ever feel as if you are under the control of some force or power other than yourself? |
| pdi_2 | Do you ever feel as if you are a robot or zombie without a will of your own? |
| pdi_3 | Do you ever feel as if you are possessed by someone or something else? |
| pdi_4 | Do you ever feel as if your feelings or actions are not under your control? |
| pdi_5 | Do you ever feel as if someone or something is playing games with your mind? |
| pdi_6 | Do you ever feel as if people seem to drop hints about you or say things with a double meaning? |
| pdi_7 | Do you ever feel as if things in magazines or on TV were written especially for you? |
| pdi_8 | Do you ever think that everyone is gossiping about you? |
| pdi_9 | Do you ever feel as if some people are not what they seem to be? |
| pdi_10 | Do things around you ever feel unreal, as though it was all part of an experiment? |
| pdi_11 | Do you ever feel as if someone is deliberately trying to harm you? |
| pdi_12 | Do you ever feel as if you are being persecuted in some way? |
| pdi_13 | Do you ever feel as if there is a conspiracy against you? |
| pdi_14 | Do you ever feel as if some organization or institution has it in for you? |
| pdi_15 | Do you ever feel, as if someone or something is watching you? |
| pdi_16 | Do you ever feel as if you have special abilities or powers? |
| pdi_17 | Do you ever feel as if there is a special purpose or mission to your life? |
| pdi_18 | Do you ever feel as if there is a mysterious power working for the good of the world? |
| pdi_19 | Do you ever feel as if you are or destined to be someone very important? |
| pdi_20 | Do you ever feel that you are a very special or unusual person? |
| pdi_21 | Do you ever feel that you are especially close to God? |
| pdi_22 | Do you ever think that people can communicate telepathically? |
| pdi_23 | Do you ever feel as if electrical devices such as computers can influence the way you think? |
| pdi_24 | Do you ever feel as if there are forces around you which affect you in strange ways? |
| pdi_25 | Do you ever feel as if you have been chosen by God in some way? |
| pdi_26 | Do you believe in the power of witchcraft, voodoo, or the occult? |
| pdi_27 | Are you often worried that your partner may be unfaithful? |
| pdi_28 | Do you ever think that you smell very unusual to other people? |
| pdi_29 | Do you ever feel as if your body is changing in a peculiar way? |
| pdi_30 | Do you ever think that strangers want to have sex with you? |
| pdi_31 | Do you ever feel that you have sinned more than the average person? |
| pdi_32 | Do you ever feel that people look at you oddly because of your appearance? |
| pdi_33 | Do you ever feel as if you had no thoughts in your head at all? |
| pdi_34 | Do you ever feel as if your insides might be rotting? |
| pdi_35 | Do you ever feel as if the world is about to end? |
| pdi_36 | Do your thoughts ever feel alien to you in some way? |
| pdi_37 | Have your thoughts ever been so vivid that you were worried other people would hear them? |
| pdi_38 | Do you ever feel as if your own thoughts were being echoed back to you? |
| pdi_39 | Do you ever feel as if your thoughts were blocked by someone or something else? |
| pdi_40 | Do you ever feel as if other people can read your mind? |
| asi_1 | Do certain trivial things ever suddenly seem especially important or significant to you? |
| asi_2 | Do you sometimes feel like you are on the verge of something really big, but you’re not sure what it is? |
| asi_3 | Do your senses sometimes seem sharpened? |
| asi_4 | Do you ever feel like you are rapidly approaching the height of your intellectual powers? |
| asi_5 | Do you sometimes notice small details that you have not noticed before that seem important? |
| asi_6 | Do you sometimes feel like it is important for you to figure something out, but you’re not sure what it is? |
| asi_7 | Do you ever go through periods where you feel especially religious or mystical? |
| asi_8 | Do you ever have difficulty telling if you are thrilled, freightened, pained, or anxious? |
| asi_9 | Do you ever go through periods of heightened awareness? |
| asi_10 | Do you ever feel the need to make sense of seemingly random situations or occurrences? |
| asi_11 | Do you sometimes feel like you are finding the missing piece to a puzzle? |
| asi_12 | Do you sometimes feel that you can hear with a greater clarity? |
| asi_13 | Do you sometimes feel like you are an especially spiritually evolved person? |
| asi_14 | Do normally trivial observations sometimes take on an ominous significance? |
| asi_15 | Do you go through periods in which songs sometimes seem to have an important meaning for your life? |
| asi_16 | Do you sometimes attribute importance to objects which you normally would not? |
| asi_17 | Do you sometimes feel like you are on the verge of figuring out something really big or important, but you aren’t sure what it is? |
| asi_18 | Has your sense of taste ever seemed more acute? |
| asi_19 | Do you ever feel like the mysteries of the universe are revealing themselves to you? |
| asi_20 | Do you go through periods in which you feel overstimulated by things or experiences that are normally manageable? |
| asi_21 | Do you often become fascinated by the little things around you? |
| asi_22 | Do your senses ever seem extremely strong or clear? |
| asi_23 | Do you ever feel like a whole world is opening up to you? |
| asi_24 | Do you ever feel that your boundaries between inner and outer sensations have been removed? |
| asi_25 | Do you sometimes feel like the world is changing and you are searching for an explanation? |
| asi_26 | Do you ever have a feeling of inexpressible urgency, and you are not sure what to do? |
| asi_27 | Have you sometimes become interested in people, events, places, or ideas that normally would not make an impression on you? |
| asi_28 | Do your thoughts and perceptions ever come faster than can be assimilated? |
| asi_29 | Do you sometimes notice things that you haven’t noticed before that take on a special significance? |
| caps_1 | Do you ever notice that sounds are much louder than they normally would be ? |
| caps_2 | Do you ever sense the presence of another being, despite being unable to see any evidence ? |
| caps_3 | Do you ever hear your own thoughts repeated or echoed ? |
| caps_4 | Do you ever see shapes, lights or colours even though there is nothing really there ? |
| caps_5 | Do you ever experience unusual burning sensations or other strange feelings in or on your body ? |
| caps_6 | Do you ever hear noises or sounds when there is nothing about to explain them ? |
| caps_7 | Do you ever hear your own thoughts spoken aloud in your head, so that someone near might be able to hear them ? |
| caps_8 | Do you ever detect smells which don’t seem to come from your surroundings ? |
| caps_9 | Do you ever have the sensation that your body, or a part of it, is changing or has changed shape ? |
| caps_10 | Do you ever have the sensation that your limbs might not be your own or might not be properly connected to your body? |
| caps_11 | Do you ever hear voices commenting on what you are thinking or doing ? |
| caps_12 | Do you ever feel that someone is touching you, but when you look nobody is there ? |
| caps_13 | Do you ever hear voices saying words or sentences when there is no-one around that might account for it ? |
| caps_14 | Do you ever experience unexplained tastes in your mouth ? |
| caps_15 | Do you ever find that sensations happen all at once and flood you with information ? |
| caps_16 | Do you ever find that sounds are distorted in strange or unusual ways ? |
| caps_17 | Do you ever have difficulty distinguishing one sensation from another ? |
| caps_18 | Do you ever smell everyday odours and think that they are unusually strong ? |
| caps_19 | Do you ever find the appearance of things or people seems to change in a puzzling way, e.g. distorted shapes or sizes or colour ? |
| caps_20 | Do you ever find that your skin is more sensitive to touch, heat or cold than usual ? |
| caps_21 | Do you ever think that food or drink tastes much stronger than it normally would ? |
| caps_22 | Do you ever look in the mirror and think that your face seems different from usual ? |
| caps_23 | Do you ever have days where lights or colours seem brighter or more intense than usual ? |
| caps_24 | Do you ever have the feeling that of being uplifted, as if driving or rolling over a road while sitting quietly ? |
| caps_25 | Do you ever find that common smells sometimes seem unusually different ? |
| caps_26 | Do you ever think that everyday things look abnormal to you ? |
| caps_27 | Do you ever find that your experience of time changes dramatically ? |
| caps_29 | Do you ever notice smells or odours that people next to you seem unaware of ? |
| caps_30 | Do you ever notice that food or drink seems to have an unusual taste ? |
| caps_31 | Do you ever see things that other people cannot ? |
| caps_32 | Do you ever hear sounds or music that people near you don’t hear ? |

*Note.* PDI = Peters Delusion Inventory (Peters et al., 1999), ASI = Aberrant Salience Inventory (Cicero et al., 2010) , CAPS

## **Results of exploratory factor analysis of PLE**

We acknowledge that the PLE score comprises overlapping constructs covered by PDI, ASI and CAPS. Therefore, we conducted a factor analysis to distinguish and cluster the items of all three questionnaires to coherent scales, allowing us to examine the sub-components of PLE more closely. Characteristics of the factor solution are presented in Table 4. Whereas the scores on factors 1, 2, 4 and 5 were associated with the βPE, only the factors 2) *Delusional ideation and auditory alteration* and 5) *Distorted bodily experience & external control* were associated with βCPP (Figure 4).

**Supplementary Table 9.** Factor analysis of identified factor structure including Cronbach’s alpha, number of items, number of cross-loadings, factor loadings, item communalities and explained variance

| Factor | Interpretation | Cronbach’s alpha | Number of items | Factor loadings | Explained variance |
| --- | --- | --- | --- | --- | --- |
| 1 | Aberrant Salience | .81 [.78, .84] | 24 | *M* = 0.53  (*SD* = 0.15) | 25% |
| 2 | Delusional and auditory alteration | .71 [.66, .76] | 20 | *M* = 0.45  (*SD* = 0.09) | 19% |
| 3 | Tactile, gustatory, olfactory alteration | .73 [.68, .77] | 16 | *M* = 0.47  (*SD* = 0.16) | 16% |
| 4 | Religious and grandiose belief | .71 [.65, .76] | 10 | *M* = 0.55  (*SD* = 0.19) | 13% |
| 5 | Distorted body experience and external control | .59 [.52, .66] | 7 | *M* = 0.48  (*SD* = 0.12) | 10% |

*Note.* 95% confidence interval given in brackets.


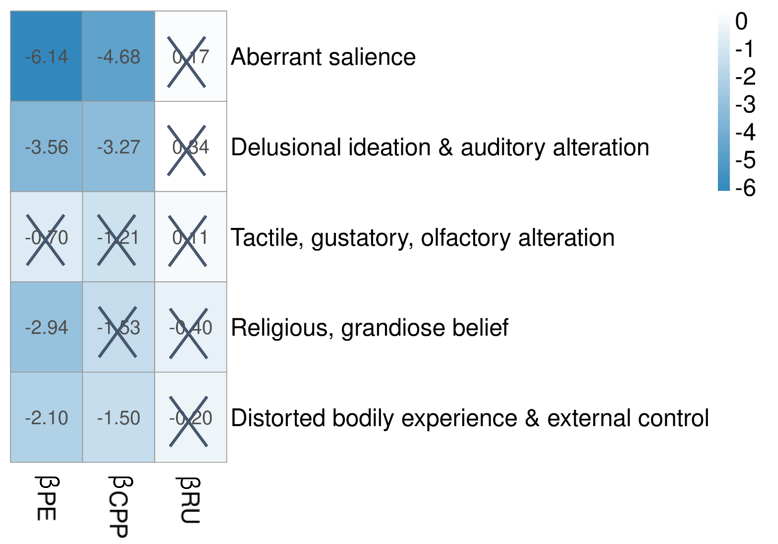


**Supplementary Figure 4.** Weight-matrix of the regression predicting individual factor scores from the exploratory factor analysis (crossed cells *p* <.05) by individual coefficients from the first-level Bayesian model regression.

**Histograms of self-reports**


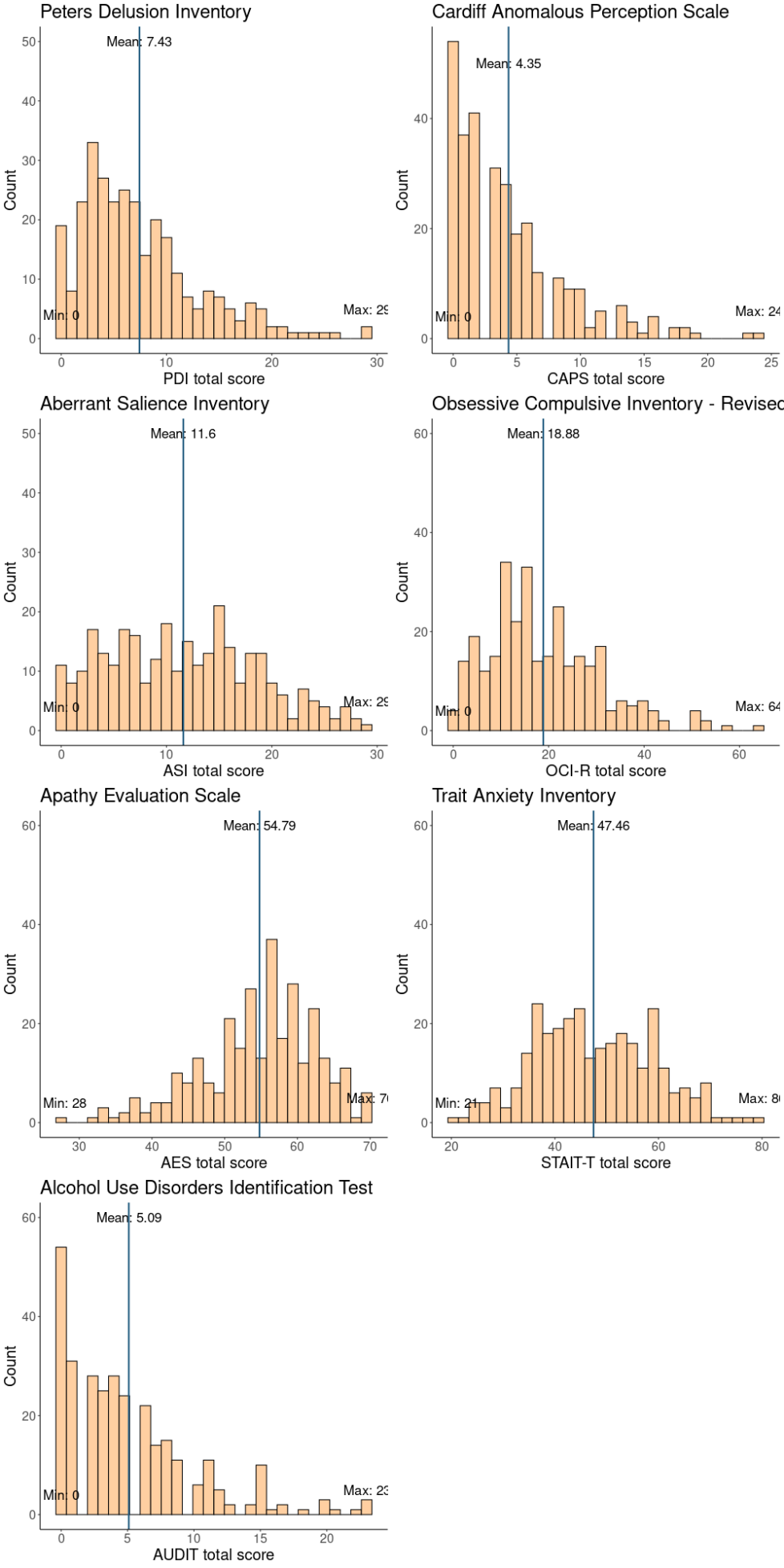


**Supplementary Figure 5**. Frequency distribution of self-report data of n = 300 participants. Vertical lines mark the group mean.

## **Histogram of model parameters**


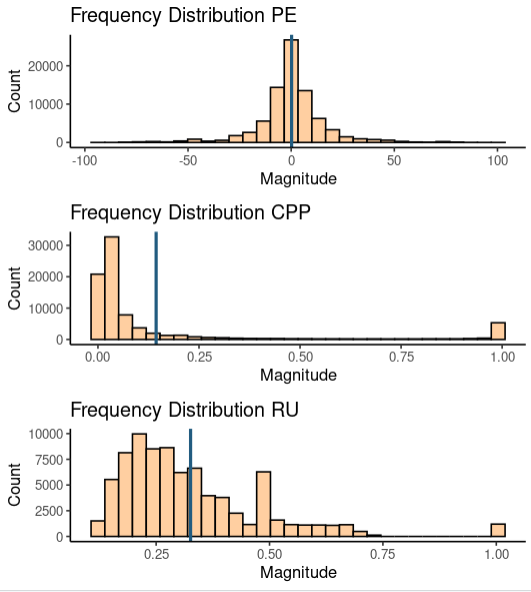


**Supplementary Figure 6.** Frequency distribution of PE, CPP and RU of all subjects n = 300 and all trials n = 280. Vertical lines mark the group mean.
